# Production and Characterization of Silk Fibroin–*Aloe vera* Hydrogel: A Study on Extraction, Hydrogel Properties, and Release Mechanism

**DOI:** 10.1021/acsomega.4c08193

**Published:** 2024-12-13

**Authors:** Camila Lopes Rodrigues, Bruno Thorihara Tomoda, Juliane Viganó, Anna Rafaela Cavalcante Braga, Mariana Agostini de Moraes, Priscilla Carvalho Veggi

**Affiliations:** a Department of Chemical Engineering, Institute of Environmental, Chemical and Pharmaceutical Sciences, Universidade Federal de São Paulo, Diadema,SP 09913-030,Brazil; b Faculdade de Zootecnia e Engenharia de Alimentos (FZEA), Universidade de São Paulo, Av. Duque de Caxias Norte 225, Pirassununga, SP 13635-900, Brasil; c School of Chemical Engineering, Universidade Estadual de Campinas, UNICAMP, Campinas, SP 13083-872, Brazil

## Abstract

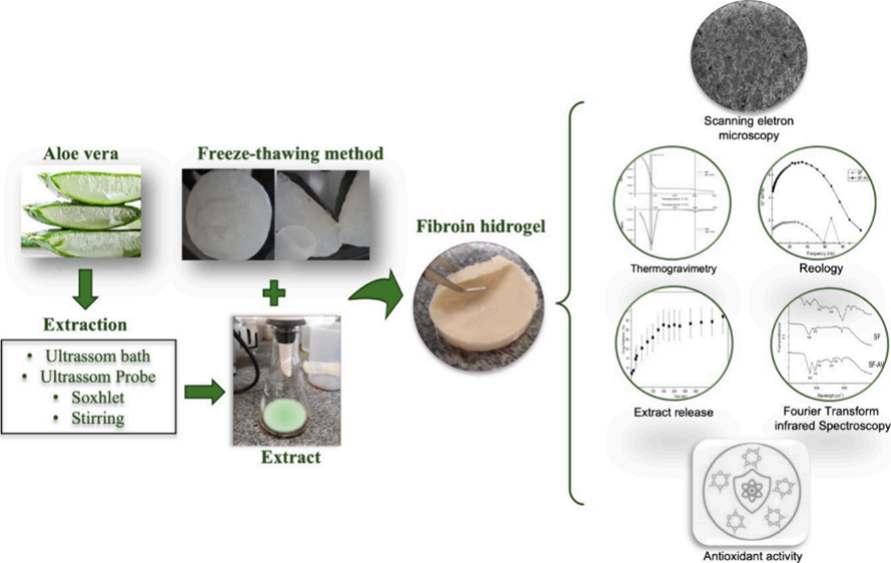

This work investigated the production and characterization of a silk fibroin (SF) hydrogel incorporated with an *Aloe vera* (AV) extract. Four extraction methods, ultrasound-assisted extraction with bath and probe, stirring, and Soxhlet, were tested, while the hydrogel was produced by a one-step freeze–thaw method. Besides the extraction yield, the antioxidant capacity of the extracts was accessed, which allowed to select the extract obtained by ultrasound-assisted extraction to be incorporated into the hydrogels. Hydrogels were characterized by scanning electron microscopy and Fourier transform infrared spectroscopy. Rheological assay, swelling behavior, and water uptake capacity were measured. The SF–AV hydrogel was submitted to release test, and the data were mathematically modeled. The hydrogels exhibited malleability, insolubility in water, interconnected pores, and thermal and physical stability. The SF–AV hydrogel released 37% extract over 330 min, with diffusion controlled by the Fickian mechanism. These promising results make the SF–AV hydrogel an attractive choice for wound dressing and other biomaterial-related applications.

## 1 Introduction

Famous for its use in the textile industry, silk fibroin is studied for applications in the biomedical area due to its biocompatibility, biodegradability, low cost, easy use, suitable mechanical properties, versatility in processing, and minimal inflammatory reaction.^1,2^ Some examples of fibroin’s multiple applications are wound dressings,^2^ controlled delivery systems,^3^ and tissue engineering.^4^ Also, fibroin can be processed in several formats, such as films, hydrogels, and nanoparticles.^2,5^

Specially, silk fibroin hydrogels have promising applications in the biomedical materials field as a wound healing dressing for having important characteristics expected for an ideal dressing.^5−7^ The sol–gel transition of a regenerated silk fibroin solution occurs naturally, but this process can take days or even weeks. Therefore, to optimize this timing process, there are plenty of methods to induce hydrogel formation.^5^ One of them is the freeze–thaw method, which creates a 3D porous structure free of metal and toxic solvents,^8^ an important parameter for applications in the pharmaceutical and medical areas.

Several studies explored alternatives to improve the fibroin hydrogel’s performance and provide even more attractive functionality associated with therapeutic compounds such as insulin, antibodies, liposomes, and sodium diclofenac.^3,9,10^ Likewise, it is promising to associate silk fibroin hydrogels with natural and green bioactive compounds such as antioxidants and healers. Kasoju and Bora^11^ encapsulated curcumin extract, a therapeutical agent in silk fibroin hydrogels establishing a new drug-release system. Results showed good interaction between fibroin and curcumin extract, in which curcumin extract’s activity was unaltered after being released from the scaffold.

In that regard, *Aloe vera* (*Aloe barbadensis* Miller) has plenty of beneficial properties, such as healing wounds and burns, anti-inflammatory, antimicrobial, anticarcinogenic, antioxidant, and antidiabetic properties, gastrointestinal benefits, and immunostimulant effect.^12−15^ According to the literature, *Aloe vera* leaves and gels are constituted by more than one hundred types of molecules, such as proteins, lipids, vitamins, and inorganic compounds.^16^ Aloin, a C-glycoside that can form aloe-emodin, is an important molecule acting as an anti-inflammatory agent, capable to reduce pain and accelerate wound healing.^12,17^ To recover these bioactive compounds, extraction processes present a feasible strategy. Extraction is one of the most used processes to obtain bioactive compounds, where a solvent acts on the plant’s cellular structure, removing the compound of interest. In this regard, the literature reports several studies concerning *Aloe vera* extraction, such as agitation, percolation, Soxhlet (conventional methods),^18,19^ and ultrasound and high-pressure solvents (nonconventional methods).^20−22^

Considering this scenario, several authors studied the association of *Aloe vera* extracts or its gel format with biopolymers such as alginate, chitosan, and silk fibroin to develop a functionalized wound dressing.^23−25^ Inpanya et al.^25^ developed an interesting film of silk fibroin and aloe gel, which proved to accelerate wound healing in diabetic rats. This study opened new possibilities for material development, associating fibroin and *Aloe vera*, which motivated the present study.

Based on the performance of the bioactive components of *Aloe vera* and the biocompatibility and versatility of silk fibroin, the development of materials with synergic association of both natural products is promising. Therefore, this study aimed to recover and select an *Aloe vera* extract with high antioxidant capacity using different methods. Afterward, a porous silk fibroin hydrogel was obtained by freeze–thaw designed to release *Aloe vera* extract. Besides that, the characterization of fibroin hydrogel containing extract and the extract release were evaluated.

## 2 Materials and Methods

### 2.1 Materials

Cocoons of the *Bombyx mori* silkworm were donated by the silk company Bratac (Londrina, Paraná, Brazil). Fresh leaves of *Aloe vera* were acquired from Biowash Comercio Importação Exportação Ltd.a (Jarinú, São Paulo, Brazil). 6-Hydroxy-2,5,7,8-tetramethylchromane-2-carboxylic acid (Trolox) and 2,2′-azobis (2-amidinopropane) dihydrochloride APPH (Sigma-Aldrich, San Luis, MO, EUA) were used to evaluate antioxidant activity (ORAC). All other analytical grade chemicals were purchased from Synth (Diadema, São Paulo, Brazil).

### 2.2 *Aloe vera* Preparation and Characterization

*Aloe vera* leaves were wrapped in black plastic bags to avoid degradation by light and stored in a domestic freezer (−18 °C) until their preparation. Then, the leaves were left to soak for 30 min in 1% (v/v) sodium hypochlorite, washed with abundant water, and then distilled water.^26^ The leaves were cut into small pieces of about 0.5 cm, distributed in Petri dishes, and stored in a domestic freezer (−18 °C) (Freezer model CRD37, Consul, Brazil) for 24 h. The frozen leaves were lyophilized (Liotop, model L101, São Carlos, SP) at a temperature of −55 °C for 72 h. The dried product was ground using a food multiprocessor, and the bigger parts were separated and milled in a knife mill (Solab Cientfica, model SL-31, São Paulo, SP). The *Aloe vera* powder was stored in amber flasks and kept in a domestic freezer at −18 °C until extraction. The mean particle size of the raw material was 0.31 ± 0.05 mm, determined using the sieving method on Tyler series sieves of 24, 32, 42, and 115 mesh (Bronzinox, São Paulo, SP, Brazil). The moisture content of the fresh *Aloe vera* leaf and powder was characterized using the AOAC method,^27^ obtaining 99.200 ± 0.070 and 6.100 ± 0.087%, respectively.

### 2.3 Extraction Processes

#### 2.3.1 Ultrasound-Assisted Extraction

The extraction procedures were conducted in an ultrasonic bath and probe. The ultrasound-assisted extraction with bath (Unique, USC-1400, Indaiatuba, SP) was previously described by Hu et al.^28^ About 1 g of raw *Aloe vera* powder was added to 50 mL of ethanol in an extraction vessel, and the mixture was sonicated for 30 min. The filtrate was evaporated by a rotary evaporator (Heidolph, Hei-VAP Precision, Schwabach, Germany) under vacuum at 60 °C. The extracts were stored in amber glass bottles at −18 °C for further steps. The assays were performed in triplicate.

The ultrasound-assisted extractions with a probe system (Sonics & Materials INC, VC505, Newtown, United States), equipped with a 1/2″ diameter probe, were performed at 500 W nominal power at 20 kHz frequency as described by Jawade and Chavan.^21^ The solution containing 7.5 g of *Aloe vera* and 100 mL of ethanol was sonicated for 30 min, keeping constant the ultrasound amplitude of 30%. After extraction, the extracts were vacuum filtered and the solvent evaporated by a rotary evaporator (Heldolph, Hei-VAP Precision, Schwabach, Germany) under vacuum at 60 °C. The extracts were packed in amber glass bottles and stored at −18 °C for further steps. The extractions were performed in triplicate.

#### 2.3.2 Stirring

The extractions were performed according to Kim et al.^18^ with some modifications. A heating magnetic stirrer was used at 60 °C using a magnetic bar inside the beakers (Lab1000, LM-MS-H280-Pro, Connecticut, USA). Extraction was carried out using 1 g of *Aloe vera* for 46 mL of ethanol 25% (v/v) as a solvent for 1 h. After extraction, the mixture was centrifuged (Eppendorf, Centrifuge 5810 R, Hamburgo, Germany) at 4 °C, 9000 rpm for 10 min. Then, the decanted solid was washed with a bit of solvent and subjected to centrifugation again. The supernatant extracts from each step were stored in amber flasks and taken to the freezer at −18 °C. To recuperate the extracts, first, supernatants were taken to rotary evaporation to remove ethanol at 60 °C, 370 mbar, and 50 rpm. Then, they were frozen and taken to the freeze-dryer for 70 h to remove water. The extracts were packed in amber glass bottles at −18 °C for further steps. The assays were performed in triplicate.

#### 2.3.3 Soxhlet

Extractions using the Soxhlet were performed following the methodology of Wang et al.^19^ Extractions were carried out in a 250 mL capacity round-bottom flask using approximately 7 g of the raw material immersed in filter paper and inserted into the Soxhlet apparatus containing 140 mL of ethanol P.A. The system was kept under reflux for 6 h. After extraction, the filtrate was removed under reduced pressure with controlled temperature by a rotary evaporator (Heidolph, Hei-VAP Precision, Schwabach, Germany) under a vacuum at 60 °C. The extracts were packed in amber glass bottles and stored at −18 °C for further steps. The extractions were performed in triplicate.

### 2.4 Extract Evaluation

#### 2.4.1 Global Yield (*X*_0_)

The global extraction yield (*X*_0_) was calculated as the mass ratio between dried extract (*m*_extract_) and dry sample (*m*_rawmaterial_), as shown in eq 1.
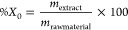
1

#### 2.4.2 Oxygen Radical Absorbance Capacity

The oxygen radical absorbance capacity (ORAC) was determined according to Murador et al.^29^ First, each extract was mixed with 100 mL of cold 80% acetone with the aid of a magnetic stirrer for 15 min. After that time, vacuum filtration was carried out, and the solids retained on the filter were washed twice with 100 mL of 80% acetone. The mixture was concentrated on a rotary evaporator. After preparation of the extracts in their hydrophilic state, the antioxidant activity against the peroxyl radical (ROO) was determined according to the ORAC test. The ROO- radical was obtained by thermal decomposition of AAPH at 37 °C. The assay was then performed in a 96-well plate, and the wells contained: fluorescein (61 nm), which was prepared in 75 mM phosphate buffer pH 7.4; AAPH solution (19 mM) in phosphate buffer; hydrophilic extract in three different dilutions (100, 500, and 1000 times) in phosphate buffer or Trolox (50 μM). The results were expressed in μmol of Trolox equivalent per gram of sample.

### 2.5 Preparation of Silk Fibroin (SF) Solution

First, sericin present on silkworm cocoons of *Bombyx mori* was extracted. Initially, the cocoons were washed with distilled water and placed in a glass flask. Then, 25 g of cocoons were degummed with 300 mL of 1 g/L Na_2_CO_3_ aqueous solution, at 85 °C, three times for 30 min each, and each time, the solution was thrown away to remove the sericin proteins. Additionally, the cocoons were washed for the last time with distilled water. After drying, 10 g of degummed fibroin fibers was cut into small pieces and dissolved in 100 mL of ternary solvent of CaCl_2_, distilled water, and ethanol on the 1:2:8 molar ratio, respectively. The solution was prepared at 85 °C, and the procedure took 90 min. The 10% (w/v) silk fibroin (SF) solution was then dialyzed against ultrapure water, using a cellulose acetate membrane (3.5 kDa MWCO, ThermoScientific) for 3 days, at 8 °C. The dialysis water was changed every 24 h, and the ratio fibroin solution:water was 1:15.

### 2.6 Preparation of Freeze–Thaw Silk Fibroin Hydrogel Containing *Aloe vera* Extract (SF–AV)

The dialyzed fibroin solution was mixed with 70% (v/v) ethanolic *Aloe vera* extract solution using a magnetic stirrer in the 3:1 (v/v) volume proportion followed by freezing for 24 h to obtain the SF–AV hydrogel. The final mass of the *Aloe vera* extract was calculated to be 0.05% (w/v) in the mixed solution. This *Aloe vera* extract concentration was based on the study of Inpanya et al.,^25^ which proved this concentration to be adequate to accelerate wound healing in diabetic rats.

After incorporation, the mixture was placed on a 6 cm diameter Petri dish and taken to a freezer at −18 °C (Freezer model CRD37, Consul, Brazil) for 24 h. Then, the Petri dish with SF–AV was placed on a dark recipient for the thawing process for 1 h at room temperature (∼23 °C). For comparison, an extract-free SF hydrogel was prepared following the same procedure: 70% ethanol was added to the SF dialyzed solution, frozen for the same period, and thawed.

### 2.7 Characterization

#### 2.7.1 Scanning Electron Microscopy (SEM)

Samples of SF and SF–AV were frozen instantly using liquid nitrogen, fractured, and lyophilized (Liotop, model L101, São Carlos, São Paulo, Brazil) for 24 h. Then, the samples were covered with a very thin layer of gold and placed in aluminum stubs. Next, the scanning electron microscope (JSM-6610LV, JEOL, São Paulo, Brazil) was set to an accelerating voltage of 10 kV, and the morphology of the hydrogels was observed.

#### 2.7.2 Fourier Transformed Infrared Spectroscopy (FTIR)

The structural characterization of the SF and SF–AV hydrogels was determined using Fourier transformed infrared spectroscopy (FTIR), in the range of 500 to 4000 cm^–1^ (Agilent Cary 630, CA, United States), on ATR mode, with 4 cm^–1^ resolution and 128 scans.

#### 2.7.3 Swelling Behavior and Water Uptake Capacity

To analyze hydrogel’s swelling and water uptake capacity, pieces of 1 cm × 1 cm dimensions of hydrogels SF and SF–AV were dried in an air circulation oven (Ethik Technology, 400–4DN, Vargem Grande Paulista, Brazil) at 40 °C for 24 h. The dried samples were left for 48 h in a desiccator with a relative humidity of 50%. Then, the samples were weighted (*W*_d_) and immersed in 4 mL of distilled water for 24 h. Finally, the samples were weighed again to obtain their swollen mass (*W*_s_). The degree of swelling and water uptake capacity of hydrogels were determined using eqs 2 and 3.
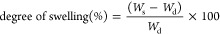
2
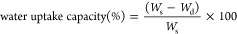
3

#### 2.7.4 Rheological Assay

SF and SF–AV rheological assays were carried out on an MCR92 rheometer (Anton Paar, Austria), with a flat plate with 20 mm diameter and 1 mm gap. The tests were performed at room temperature (23 °C) in duplicate. The samples of each hydrogel were prepared in a cylindrical mold, were approximately 100 mm in diameter and 80 mm in height, and were cut to approximately 10 mm in thickness.

First, a tension scan of each sample was performed to obtain the linear viscoelastic region. After that, the voltage found in each sample was used in the frequency variation tests, which took place in an interval from 1 to 100 Hz.

#### 2.7.5 Thermogravimetric Analysis (TGA)

Thermal properties of the SF and SF–AV hydrogels were determined by thermogravimetric analysis. The heating rate used was 10 °C/min under a 50 mL/min nitrogen atmosphere in the temperature range of 25 to 600 °C.

### 2.8 Release Assay

SF–AV hydrogels were placed in a beaker with 150 mL of phosphate-buffered saline (PBS) at 37 °C under constant slight agitation. Over time, the supernatant was measured by UV/vis spectroscopy (Thermo Fisher Scientific, Waltham, MA, EUA) at 297 nm, which is attributed to one of the aloin’s peaks.^30^ Each aliquot analyzed was returned to the beaker. Analysis was performed in triplicate until an equilibrium was established. The amount of released extract as a function of time was determined based on the standard extract curve in PBS.

### 2.9 Mathematical Modeling

The kinetic data were fitted using different mathematical models using Microsoft Excel and Origin Pro 8.0. Peppas model (eq 4) is known as a semiempiric model,^31^ where  is the fraction of the drug released at time *t* (s), *k* represents the diffusion coefficient, and *n* indicates the predominant release mechanism.
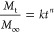
4

The value of *n* depends directly on the geometry of the release matrix, and it is an indication of which mechanism is predominant in the release system.^31,32^

When n is less than or equal to 0.5 for thin films and less than or equal to 0.45 and 0.43 for cylinders and spheres, respectively, the release mechanism of the evaluated system is controlled by Fickian diffusion. If the exponent is 1, 0.89, and 0.85 for thin films, cylinders, and spheres, respectively, it indicates the transport case-II mechanism. If the exponent is in the intermediate range, it has an anomalous release; that is, its release is a phenomenon that involves the superposition of swelling and diffusion.^31^ Some variations of the Peppas model are also analyzed, such as the zero order model, in which *n* equals 1 (eq 5), and the Higuchi Model, in which *n* equals 0.5 (eq 6).^15^
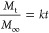
5
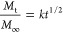
6

Another model used to analyze the release kinetics was the Peppas–Sahlin model (eq 7), applicable in anomalous release systems in which Fickian diffusion is not the only phenomenon that controls the release. It considers *m* as the exponent related to the mechanism of drug release, the Fickian diffusion contribution is observed on the first part of the equation, described by kinetics constant *k*_1_, and the transport case-II is observed on the second part, described by kinetics constant *k*_2_.^33^
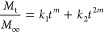
7

### 2.10 Statistical Analysis

Experimental results were statistically analyzed using Minitab 18 software, using Tukey test with 95% of confidence, so differences were considered statistically significant at a significance level of *p* < 0.05.

## 3 Results and Discussion

### 3.1 Extract Characterization

**Figure 1:**
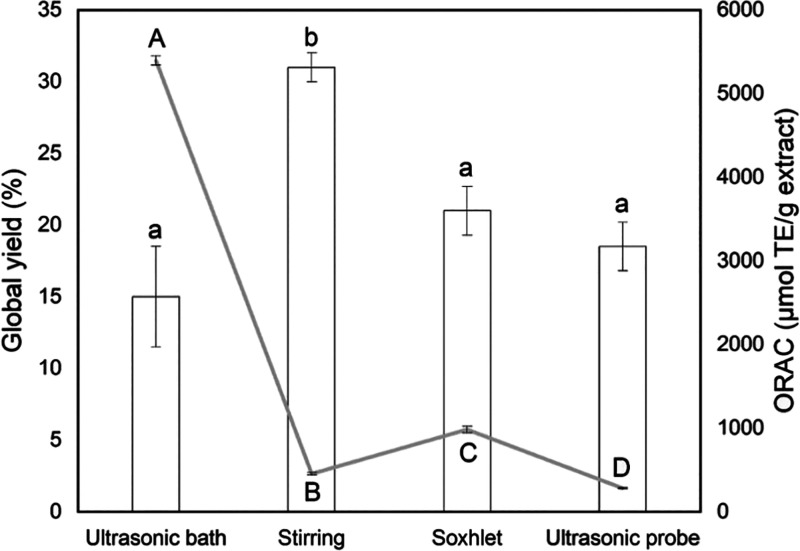
Effect of the extraction method on the global yield and antioxidant capacity for *Aloe vera* extracts. Different small letters between columns indicate significant difference (*p* < 0.05) regarding a global yield, and different capital letters indicate significant difference (*p* < 0.05) regarding the antioxidant capacity.

Figure 1 presents the results for the global extraction yield (*X*_0_) as columns and antioxidant capacity (ORAC) as lines obtained under different extraction methods on a dry basis. According to the results, extraction with water in the solvent allowed us to obtain high *X*_0_ by stirring (31.0 ± 1.0%), since it was the only method in which water was used as the solvent (25% v/v, ethanol in water). Kim et al.^18^ optimized the extraction process of the lyophilized powder from the *Aloe vera* gel. They proved that the ethanol–water ratio directly interferes with the extraction yield, in which a high ethanol concentration (greater than 90%) resulted in lower *X*_0_. The same behavior was observed in the present work for extraction using ethanol in the ultrasound-assisted extraction with bath (15.0 ± 3.5%), Soxhlet (21.0 ± 1.7%), and ultrasound-assisted extraction with probe (18.5 ± 1.7%). However, the extraction yield does not necessarily indicate the effectiveness of the extract, which is related to the concentration of bioactive compounds.

*Aloe vera* has several antioxidant components presenting different polarities.^15,18^ Therefore, in choosing the ideal solvent, it is necessary to determine whether the target compound is soluble. The high extraction yield of the stirring process suggests that the obtention of water-soluble compounds, such as carbohydrates, of high or low molecular weight, contributes to the high yield^34^ but did not add antioxidant capacity to the extract. The extract obtained by stirring was the only extract that presented a dark pink color after recovery. According to Suriati^35^ and Asif et al.,^36^ the darkening of *Aloe vera* can result from the oxidation of carbohydrates in its composition, affected, for example, by exposure to oxygen or high temperatures.

Soxhlet, ultrasonic probe, and ultrasonic bath showed lower extraction yields, but this does not mean that these extracts have lower levels of active compounds. There was a significant disparity in the antioxidant capacity between the ultrasonic bath and the other methods, the ultrasonic bath being the most suitable, therefore, for obtaining an extract with a higher concentration of antioxidant compounds. It was found 5396.2 ± 55.8 (μmol TE/g extract) for ultrasonic bath and 282.7 ± 7.2, 454.5 ± 15.1 and 983.2 ± 38.1 (μmol TE/g extract) for ultrasonic probe, stirring, and Soxhlet, respectively. Such results may be explained by the characteristics of the extraction methods; ultrasound-assisted extraction with a probe displays a huge energy in the extraction medium that may affect the stability of the compounds by increase in the temperature and acoustic cavitation, while the extraction in the Soxhlet apparatus maintains the extract for a long time exposed to the solvent boiling temperature that may affect its stability and bioactivity. According to the literature, long periods at high temperatures can decrease the *Aloe vera* compound activity.^37,38^ Therefore, concerning its high antioxidant activity, the *Aloe vera* extract prepared by ultrasound-assisted extraction in a bath was selected to be incorporated in fibroin hydrogel.

Concerning the ultrasonic bath, Hu et al.^28^ obtained *X*_0_ of 6% extracting the *Aloe vera* leaf and gel. Wang et al.^19^ used only the *Aloe vera* bark and ethanol as a solvent using Soxhlet and found about 2.53 × 10^–4^% of aloe-emodin from *Aloe vera*. Jawade and Chavan^21^ extracted the *Aloe vera* gel applying ultrasonic probe and Soxhlet. The ultrasonic probe was optimized in terms of the solvent type (methanol, ethanol, and isopropanol). Higher *X*_0_ was obtained using methanol as a solvent, obtaining 0.0515 and 1.84%, respectively, with Soxhlet and ultrasonic batch. However, due to the environmental impact of using methanol as a hazardous waste, this study considered ethanol, which was probably reflected in the *X*_0_ results. In addition, unlike in this study, the authors studied only the *Aloe vera* gel.

There are few studies in the literature regarding extraction methods and antioxidant analysis of the whole leaf of *Aloe vera*. Some studies use only the gel, which is the inner part of the leaf.^18,21^ Kim et al.^18^ extracted the *Aloe vera* gel using the stirring method employing 34% (v/v) ethanol in water as a solvent at 60 °C for 1 h. In the study, an antioxidant analysis was performed using the DPPH and FRAP methods, obtaining concentrations of 10.9 and 49.1 μmol of TE/g of the dry sample, respectively. There were also studies regarding the extraction from the *Aloe vera* bark.^8,19,39^ Vidic et al.^39^ extracted bioactive compounds from the dry skin of *Aloe vera* by Soxhlet, using absolute ethanol as a solvent. The antioxidant analysis method DPPH reached a concentration of 45.6 IC_50_ mg/mL for the extract. The *Aloe vera* skin was also studied by Ioannou et al.^22^ The authors optimized ultrasound-assisted extraction with a probe in combination with deep eutectic solvents, finding concentrations of 64.96 ± 3.04 mg of GAE/g of the dry sample and 2217 ± 39 μM TE/g of the dry sample for TPC and FRAP, respectively.

### 3.2 Physical Aspects of Hydrogels

One of the biggest challenges of the current study was to incorporate the extract into dialyzed silk fibroin solution and perform its gelation, ensuring homogenization. The fibroin solution is thermodynamically metastable in the dialyzed form and easily performs an uncontrolled sol–gel transition.^40^ If the preparation of the SF–AV hydrogel were performed inappropriately, the incorporation of the *Aloe vera* extract would be incomplete or partially complete. Thus, several attempts were made, and alternatives were explored to obtain the best way of incorporation.

**Figure 2:**
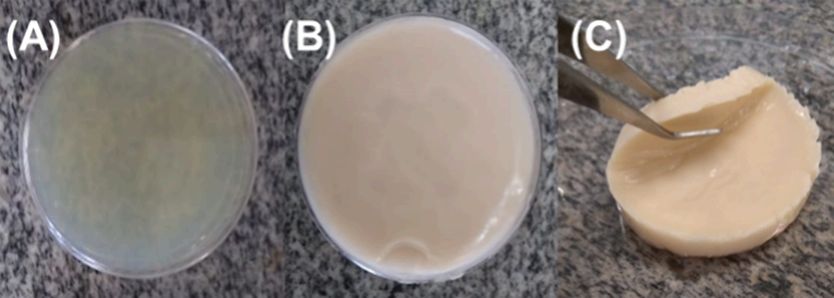
Photographs of SF–AV (A) before and (B, C) after the freeze–thaw technique.

*Aloe vera* extracts from the ultrasound bath seemed like a dark green slush. However, the regenerated silk fibroin solution became light green right after incorporation, as shown in Figure 2A. After the freeze–thaw technique, the SF–AV hydrogel exhibited a light green color, and after a few days of storage at the refrigerator (−4 °C), it showed a brown color (Figure 2B). The same behavior was observed by Khoshgozaran-Abras et al.,^41^ which incorporated *Aloe vera* gel extracts on chitosan-based films. The more extract they added to the gel, the more the films got darker, which was probably the result of the oxidation of the anthraquinones.^42^ Also, the browning process of *Aloe vera* was probably due to the carbohydrate’s oxidation, promoted, for example, by oxygen and temperature of storage.^35^ Another possible reason for the darkening may be the chlorophyll photoionization process, present in the *Aloe vera* and possibly extracted in the current study, since ethanol is one of the best solvents to extract it. This reaction replaces the magnesium in the center of the chlorophyll molecule with hydrogen. It is stimulated when the molecule is subjected to low temperatures, such as the one used in the freeze–thawing technique.^43,44^

The SF–AV hydrogels were malleable (Figure 2C) and had gelatinous consistency and water insolubility, important characteristics for wound dressings.^45^ Its malleability can provide a comfortable experience during use. The extract-free hydrogel (SF) also had the same features. Both hydrogels were fragile upon soft handling, tearing apart when not handled appropriately.

#### 3.2.1 SEM

**Figure 3:**
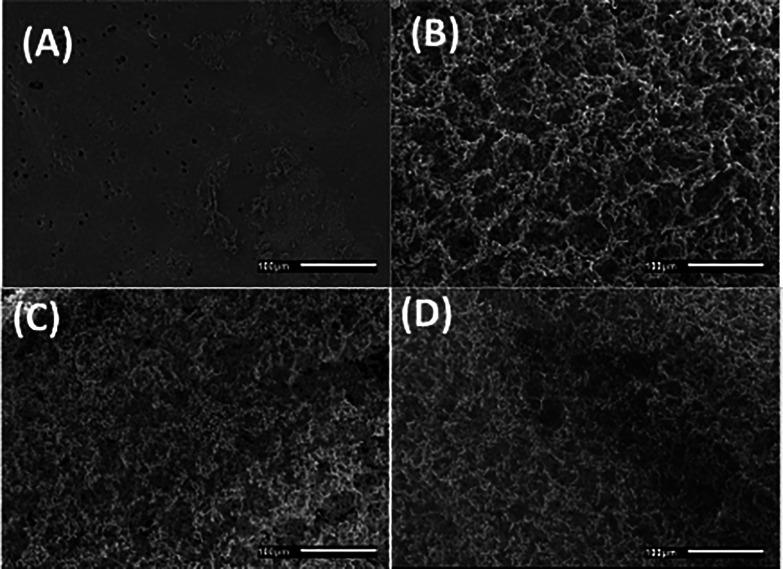
SEM surface micrographs of (A) SF–AV and (B) SF and fracture micrographs of (C) SF–AV and (D) SF.

SEM micrographs of SF–AV showed a smooth flatter surface (Figure 3A), while the *Aloe vera* free hydrogel, SF (Figure 3B), showed a rough surface. The cross-sectional micrographs also showed pore structures (Figure 3C,D), indicating the interconnected porous inner structure of SF and SF–AV hydrogels. According to the literature, this interconnected structure is very attractive to biomedical applications because it can work as the storage of molecules once it has a high surface area to absorb them and facilitate their delivery, permeation, and diffusion.^46^ In our study, the purpose of using the freeze–thaw technique was to form a porous scaffold, which was successfully achieved.

The porous structure is a consequence of the freeze–thaw method used for fibroin hydrogel preparation. During the freezing process of SF and SF–AV solution, water from SF solution and extract solution formed ice crystals excluding the solutes.^8^ Ice crystals keep growing during freezing, especially when the freezing temperature is high. In other words, higher freezing temperature results in bigger ice crystals than lower freezing temperatures.^47^ Therefore, the porous structure results from the ice nucleus formed during freezing.

When frozen, SF proteins concentrate, unfold, aggregate, and dehydrate, so pore wall composition is a highly concentrated fibroin network built during freezing, when fibroin molecules rearranged.^8,48^ At room temperature, the ice crystals present on scaffolds thawed, resulting in a silk fibroin hydrogel with a porous structure that was liquid saturated.

According to the literature, the pore size of hydrogels obtained by freezing techniques is directly dependent on the temperature of the freezing treatment and concentration of silk fibroin solution.^47,48^ In this study, the freezing process was performed on a domestic freezer (around −18 °C), resulting in a broad distribution of nonuniform pore sizes, as shown on SEM images (Figure 3). However, the freezing temperature used, around −18 °C, is considered a high freezing temperature, which may help the crystallization process.^49^

One strong advantage of the methodology used is that it does not require freeze-drying or repeated freeze–thaw cycles, just the use of a small amount of miscible organic solvent (ethanol) and only one cycle of freeze–thaw. This one-step preparation reduces the time and energy required for the hydrogel preparation. Also, this method does not use any toxic cross-linker to form the hydrogel structure, making it safe and metal free for application and use by humans.^5^ In addition, the simplicity of the freeze–thaw technique is that the incorporated extract is not exposed to too many preparation steps or chemicals that could degrade or reduce its activity.

#### 3.2.2 FTIR

**Figure 4:**
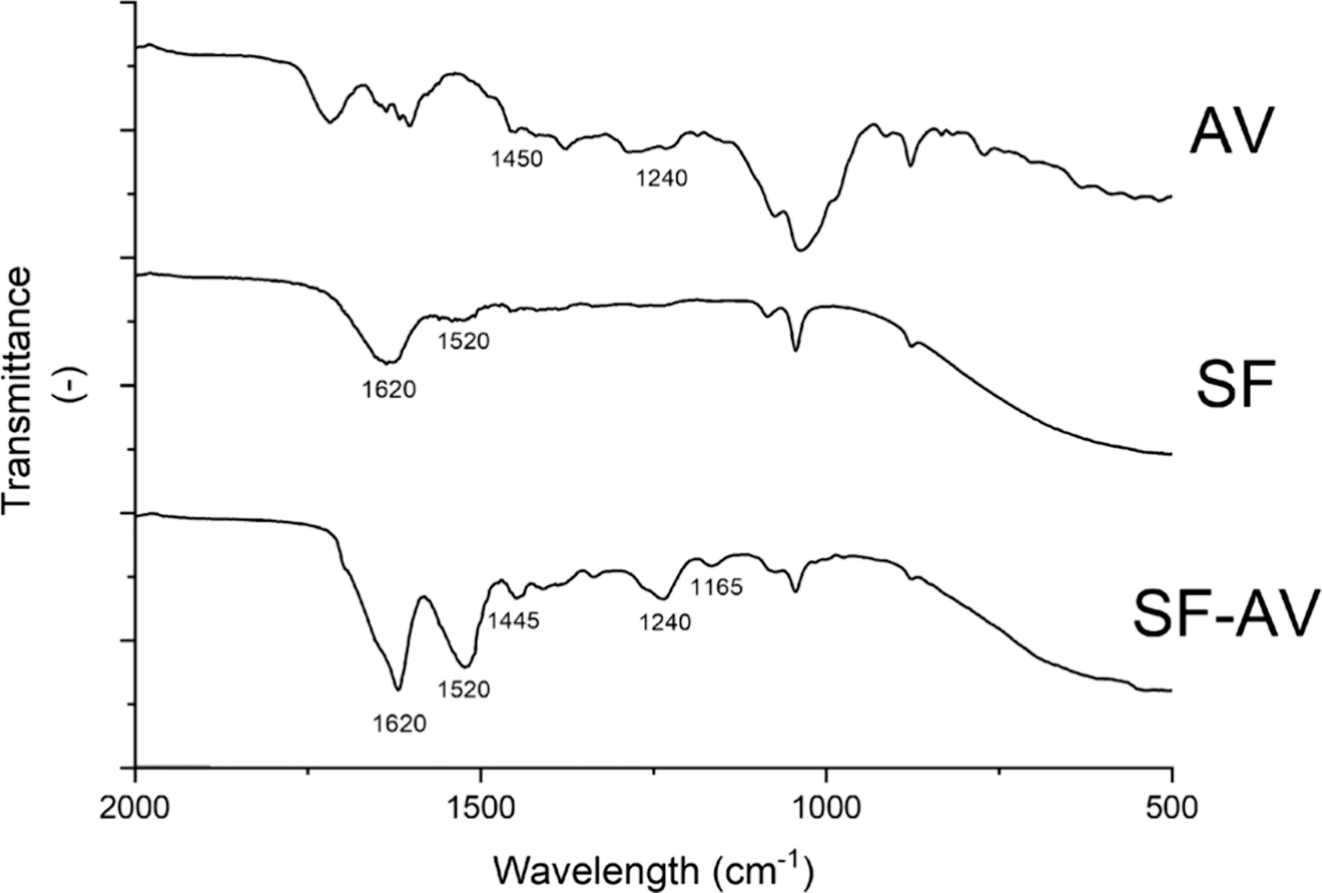
FTIR spectra of the *Aloe vera* extract (AV), silk-fibroin *Aloe vera* (SF–AV) hydrogel, and silk-fibroin (SF) hydrogel.

Both SF and SF–AV spectra presented peaks at 1620 cm^–1^ (amide I) and 1520 cm^–1^ (amide II), corresponding to silk II conformation (β-sheet).^58^ Amide I is related to C=O stretching and amide II with the N–H bending.^50^ It can be noted that amide I and II peaks are present in the spectrum of the extract-embedded hydrogel (SF–AV) at the same wavelength as the extract-free hydrogel (SF) (Figure 4). Therefore, it indicates that the presence of *Aloe vera* extract did not affect the silk fibroin conformation. Amide III peaks and peaks corresponding to the random coil conformation were not observed. However, it cannot be affirmed that random coil conformation is not present on both scaffolds: they can occur in a lower proportion than the β-sheet conformation.

It was observed in the SF–AV and AV a peak at 1240 cm^–1^. According to the literature, a band in this region is due to elongation of the CO groups, suggesting the presence of esters and phenols in both the SF–AV hydrogel and the AV extract. This peak is not present in the spectrum of the extract-free hydrogel (SF), indicating that incorporation of the extract was successful in the SF–AV hydrogel. Still, another peak was observed in SF–AV and AV spectrum, at 1445 and 1450 cm^–1^, representing the presence of COOH. The peak observed at 1165 cm^–1^ in SF–AV hydrogel is attributed to the tyrosine side chains, related to fibroin chain.^51^

No additional bands that would indicate interactions between fibroin and *Aloe vera* extracts were observed. This suggests that covalent bonds were not formed between the *Aloe vera* extract and fibroin molecules. Thus, it is possible that other types of bonds, not detectable by the FTIR equipment exist, such as electrostatic interactions, or that the extract is simply physically entrapped in the SF–AV hydrogel occupying the empty spaces of the inner, inside pores.

The sol–gel transition of a hydrogel, better known as the gelation process, results from the cross-linking of polymer chains, and it can be physical or chemical cross-linking.^52^ The β-sheet conformation and, consequently, its insolubility in water may be induced by the physical cross-linking reached by the freeze–thaw technique because it induces silk fibroin crystallization.^7^ According to Li et al.^47^ during the process of thawing, the thermal kinetic energy of chain segments increases, and to handle it, they revolve around itself to make molecules stretch, producing a partially ordered structure and, consequently, increasing crystallinity, making it hard to dissolve in water.

Additionally, the presence of ethanol on the SF and SF–AV compositions could have induced the β-sheet conformation. Adding the extract with ethanol 70% (v/v) can be considered a chemical cross-linking once it induces β-sheet conformation.^53^ In this study, ethanol was used to dissolve the AV extract. Thus, the same solvent (ethanol) was used for two goals: dissolve the extract and induce β-sheet conformation on silk fibroin and, consequently, hydrogel formation. Kasoju and Bora^11^ incorporated curcumin extract in silk fibroin solution using dimethyl sulfoxide (DMSO) and observed that adding organic solvent can induce conformational changes in fibroin at low temperatures. Oztoprak et al.^48^ also used the freeze–thaw technique and EGDE (ethylene glycol diglycidyl ether), obtaining a porous structure similar to the one observed on Figure 4.

From a thermodynamic point of view, SF random coil tends to migrate to β-sheet conformation, a more stable state, and to accomplish that an energy barrier needs to be overcome.^54^ This indicates that chemical and physical cross-linking used in our study promoted enough energy and molecular activity to SF chains, inducing intra- and intermolecular interactions and transitioning to β-sheet conformation.^40,54^

#### 3.2.3 Swelling Behavior and Water Uptake Capacity

The ability to absorb external fluids, such as the exudate of a wound, is a fundamental characteristic of an ideal wound dressing.^55^ The SF–AV hydrogels had a lower (*p* < 0.05) swelling than SF hydrogels (Table 1), showing the influence of *Aloe vera* extract incorporation. The SF surface is more porous than SF–AV, which could be seen in Figure 3, so this could be an extra entry of surrounding fluids compared with the SF–AV surface.

**Table 1:** Water Uptake Capacity and Degree of Swelling of the SF and SF–AV Hydrogels^a^

hydrogel	degree of swelling (%)	water uptake (%)
SF–AV	71.64 ± 16.29^a^	41.15 ± 5.22^a^
SF	120.56 ± 10.80^b^	54.52 ± 2.30^b^

a Different letters in the same column indicate a significant difference (*p* < 0.05).

Water uptake was also higher on SF hydrogels than SF–AV ones (*p* < 0.05). Inpanya et al.^25^ prepared silk fibroin films incorporated with *Aloe vera* extract using the solvent casting technique and reached a water uptake capacity of 43.7 ± 2.6% and a swelling ratio of 0.8 ± 0.1 g/g (ca. 80% of swelling degree), similar to our results. The values reached for degree of swelling and water uptake capacity are ideal, since a water uptake capacity higher than 80% would lead to tearing during handling.

Water uptake could be limited because of the silk fibroin conformation on both hydrogels: the silk fibroin β-sheet conformation provides resistance against relaxation and, consequently, forms highly cross-linked rigid pore walls decreasing the possibility of expansion to absorb external fluids and storage of water molecules between the polymeric chains.^23,49^ Besides that, the presence of a β-sheet conformation has positive benefits for SF–AV since it provides a higher resistance to degradation.

It was expected that both characteristics were higher on SF–AV because of the hydrophilic behavior of the *Aloe vera.*(56) However, the hydrogels with *Aloe vera* extract incorporated showed a lower capacity of swelling and absorbing fluids from the surroundings, proving that the pore size may have directly affected the swelling ability since big pores provide more space for water molecule storage.

#### 3.2.4 Rheology

**Figure 5:**
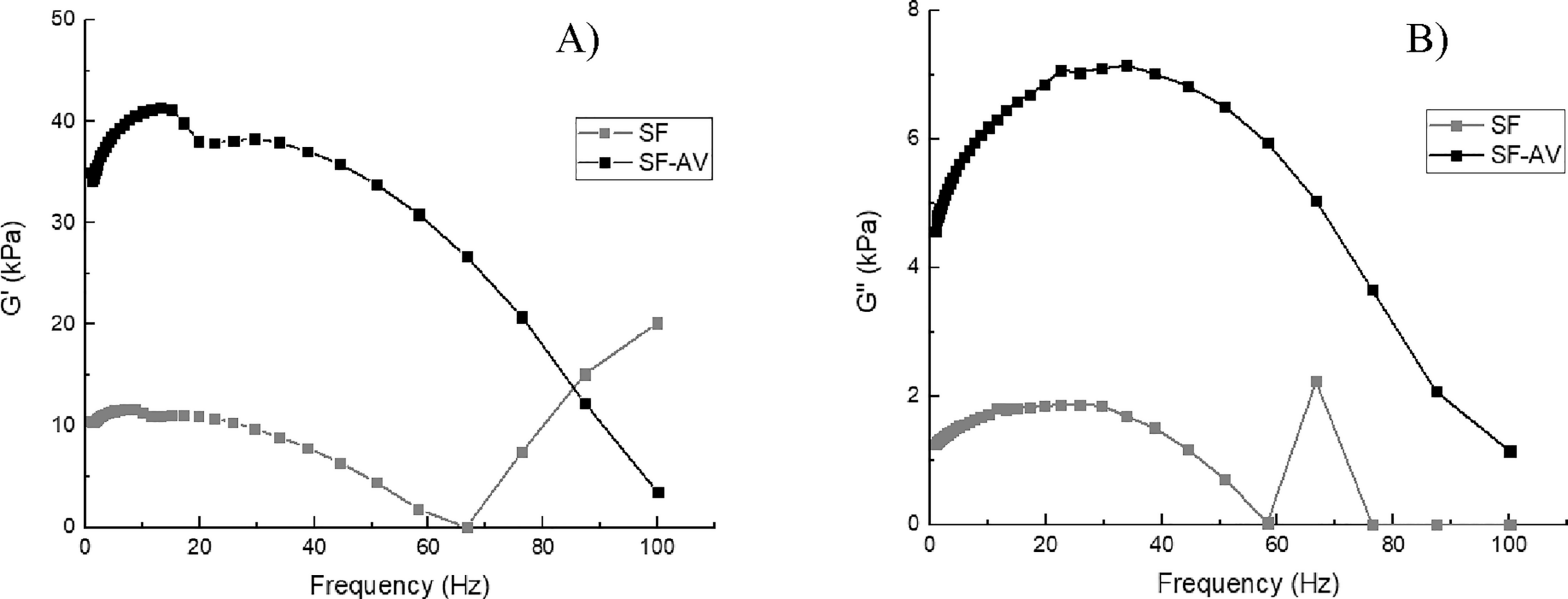
(A) Storage modulus (*G*′) and (B) loss modulus (*G*″) in the function of frequency (Hz) of SF and SF–AV hydrogel.

Rheology assays were used to investigate whether the SF–AV and SF hydrogels have viscous or elastic predominant behavior. In both hydrogels, the storage modulus *G*′ was greater than the loss modulus *G*″ (Figure 5), indicating that the polymeric network formed was well-developed.^57^ The fact that *G*′ is higher in SF–AV is directly associated with the increase in the β-sheet content and its consequent predominant elastic behavior.^58^ In addition, the *G*′ and *G*″ modulus being higher in SF–AV indicates that the presence of *Aloe vera* extract did not reduce or negatively affect the interaction among silk fibroin molecules. On the contrary, the *Aloe vera* incorporation actually improved the SF–AV hydrogel strength and elasticity. *Aloe vera* gel has an elastic behavior attributed to its network of polymeric chains.^59^ So, incorporating the *Aloe vera* extract on the fibroin hydrogel has improved the elasticity of the material.

#### 3.2.5 TGA

**Figure 6:**
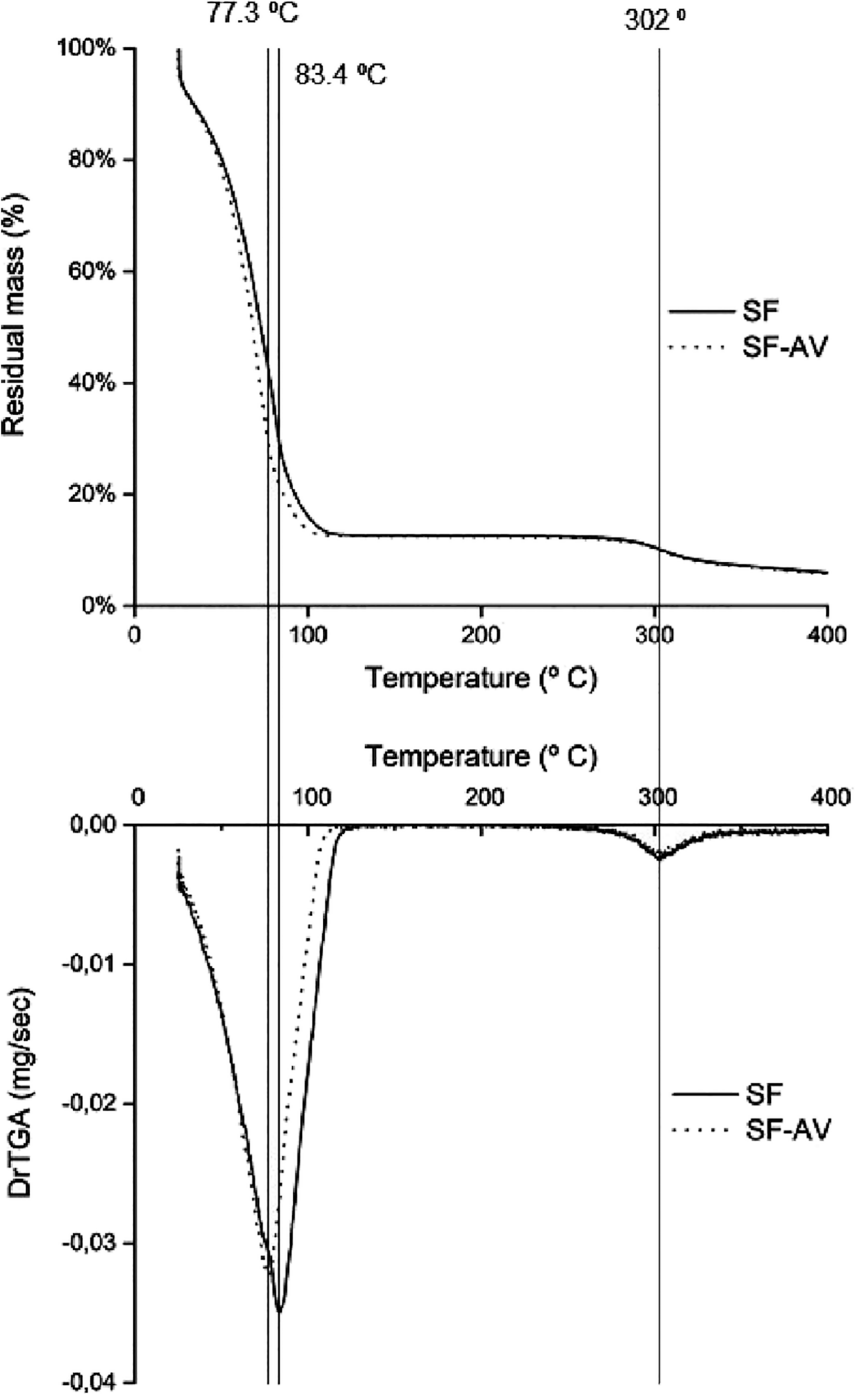
(A) Thermograph and (B) derivative thermograph of the SF and SF–AV hydrogels.

Thermogravimetric analysis (TGA) and derivative thermogravimetry analysis (DrTGA) are presented in Figure 6A,B, respectively. It was observed for both SF and SF–AV a mass loss peak at 77.3 and 83.4 °C, respectively, related to moisture loss. Another thermal event was observed at 302 °C. This peak results from the breakdown of side chain groups present on amino acid residues and the cleavage of peptide bonds. In addition, silk fibroin hydrogels with β-sheet conformation usually decompose in the 290–295 °C temperature range, and amorphous silk fibroin decomposes at temperatures below 290 °C.^53^

The similarity of SF and SF–AV thermogravimetric curves indicates that *Aloe vera* extract’s presence did not change the silk fibroin hydrogel thermal stability. Considering the potential application of SF–AV hydrogels as wound dressings, thermal events should not occur at temperatures lower than 37 °C (body temperature).^56^ Since there is no peak around this temperature range, SF–AV can be considered a thermally stable material for skin applications.

### 3.3 Release Assay

**Figure 7:**
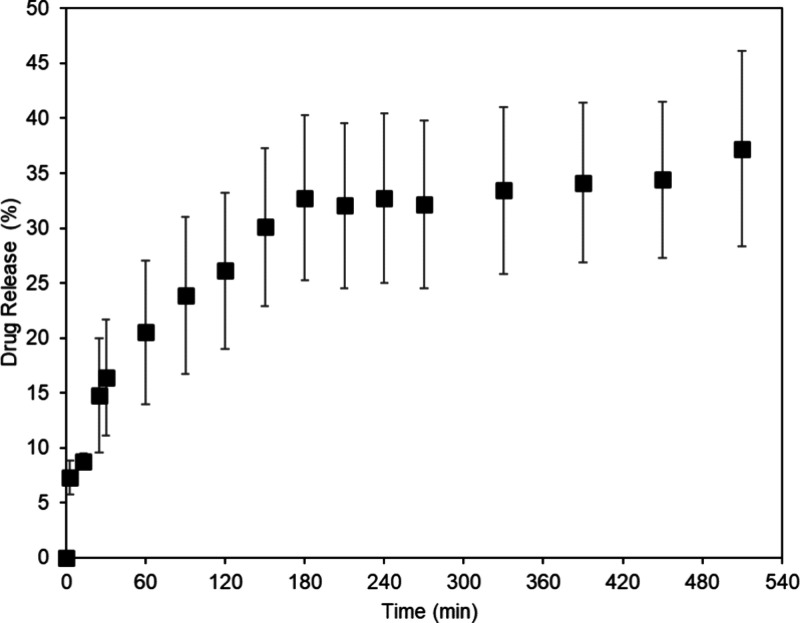
*Aloe vera* extract release profile as a function of time on the SF–AV hydrogel.

In addition to the structural and thermal characterization of SF–AV, the *Aloe vera* release from the hydrogel is critical to determine the feasibility of using the wound healing potential.^60^ The release profile of the SF–AV hydrogel can be seen in Figure 7, indicating the mass fraction released over time. To find out the release mechanism of *Aloe vera* from SF–AV hydrogels, the data of the release assay were fitted to the equations of Peppas, zero order, Higuchi, and Peppas–Sahlin (eqs 4 – 7) considering the fitting restriction to up to 60% of the total released mass (≤ 0.6).^31^ The values of the constants of the equations are listed in Table 2.

**Table 2:** Fitting Parameters of the Mathematical Models Are Assigned to the Kinetic Release Data

**model**	**parameters**		***R***^**2**^
Peppas	*k*	0.024 ± 0.011	0.971
	*n*	0.377 ± 0.059	
Higuchi	*k*	0.0096 ± 0.0005	0.947
zero order	*k*	0.0002 ± 0.000	0.566
Peppas–Sahlin	*k*_1_	0.074 ± 0.041	0.940
	*k*_2_	0.000 ± 0.008	
	*m*	0.500 ± 0.346	

It is important to highlight that the aloin peak intensity of all of the experimental points was monitored, suggesting that this compound was not degraded during the 540 min of release. Moreover, the SF hydrogel did not show any interference in the absorbance at 297 nm during the whole test, indicating that fibroin itself does not affect the spectrum reading at this wavelength.

It is possible to observe that *Aloe vera* extract release from silk fibroin hydrogel was fast, reaching equilibrium at approximately 330 min, with the maximum fraction released of 37.2 ± 8.9%. These values are similar to those of Bierhalz et al.,^23^ which observed a total amount of aloin release of 40% from chitosan–alginate membranes and of 50% release from chitosan–xanthan membranes. The authors attributed the high aloin retention in both membranes to the formation of hydrogen bonds between the polysaccharides and aloin, which has six OH groups in its structure. Also, the equilibrium of aloin release was reached in approximately 1 h for chitosan–xanthan membranes, while this time increased to 5 h for chitosan–alginate membranes, which was attributed to the high degree of cross-linking induced in the chitosan–alginate membranes. Similarly, in the present study, we can assume that aloin may have interacted with fibroin by hydrogen bonds and that the cross-linking induced by the freeze–thaw method may have influenced the release time until equilibrium.

Also, to understand the mechanisms dominating the release, fitting of mathematical models were performed to kinetic release data. The zero order model was not adequate to fit the data, as observed from the low value of regression coefficient (*R*^2^) at Table 2. The Peppas model exhibited a higher value of regression coefficient (*R*^2^), proving to be the best model to fit the release kinetic data. The coefficients *n* and *m* from Peppas and Peppas–Sahlin models indicate a major contribution from Fickian diffusion in the *Aloe vera* release.

Moreover, the constant *k*_2_, from the Peppas–Sahlin model, was zero, indicating the low swelling contribution to the release of SF–AV hydrogels. The SF–AV hydrogel is naturally swollen, having a considerable amount of water in its structure, so it is already saturated with water and does not swell when inserted in the release medium. This behavior is corroborated by the swelling results (Table 1), which showed a really small swelling index of SF and SF–AV hydrogels. Therefore, the contribution of swelling in the release is small, with a high contribution of Fickian diffusion.

Kasoju and Bora^11^ also observed Fickian diffusion as the dominant mechanism of the release of curcumin from silk fibroin hydrogels. However, the authors observed a slow and sustained release of curcumin for approximately 100 h. The authors attributed the slow release of curcumin to its hydrophobic character, which led to a low affinity with water and, at the same time, allowed the formation of hydrophobic interactions with fibroin hydrophobic cores.

The release kinetics is directly influenced by the matrix polarity and the solute to be released. *Aloe vera* high hydrophilicity can be considered a challenge, as it is highly soluble in the release medium, resulting in burst release.^61^ According to the literature, this behavior should be avoided since a prolonged, stable, and controlled release for a longer time aims to optimize the release system.^62^ For wound dressing, the longer the release time, the greater the maintenance of the concentration of therapeutic agents administered to the patient and the lower the dressing change times.^63^ In contrast, the wound has a greater chance of inflaming in the early stages. Hence, a dressing that releases therapeutic agents as soon as it comes into contact with its release medium, such as SF–AV releasing the bioactive compound can help avoid such complications.^64^

## 4 Conclusions

Extraction methods have significant holes in the extraction yield and antioxidant capacity. The method employing water in the solvent showed higher yields but lower antioxidant capacity, indicating the extraction of nonantioxidant compounds. Besides, methods that expose the extract to higher temperature and intensity acoustic cavitation also showed low antioxidant capacities. Through antioxidant capacity assay (ORAC), it was possible to conclude that the ultrasound-assisted extraction with bath was the most suitable to extract antioxidant compounds from *Aloe vera* and the resulted extract was selected to be incorporated into silk fibroin hydrogels (SF -AV).

SF–AV hydrogels were prepared by a one-step method, using the freeze–thaw technique, which resulted in hydrogels with an interconnected porous structure. The association of multiple biological properties of *Aloe vera* extracts and silk fibroin’s hydrogel resulted in malleable and water-insoluble hydrogels, which adhere smoothly to surfaces and have a viscoelastic behavior, such as human skin, important characteristics for a wound dressing. The thermal and structural analysis also showed that *Aloe vera*’s presence did not interfere with silk fibroin stability. The *Aloe vera* release from SF–AV hydrogels showed equilibrium in 330 min, and the Peppas model presents the best fitting to the release data, indicating that the release is controlled by Fickian diffusion. By analyzing the whole results, it is possible to conclude that SF–AV hydrogels represent an alternative and all-natural material to be used for potential wound healing applications.

## Data Availability

Data will be available on a reasonable request to the corresponding author.
